# Neuroimaging of Inflammation in Memory and Related Other Disorders (NIMROD) study protocol: a deep phenotyping cohort study of the role of brain inflammation in dementia, depression and other neurological illnesses

**DOI:** 10.1136/bmjopen-2016-013187

**Published:** 2017-01-05

**Authors:** W Richard Bevan-Jones, Ajenthan Surendranathan, Luca Passamonti, Patricia Vázquez Rodríguez, Robert Arnold, Elijah Mak, Li Su, Jonathan P Coles, Tim D Fryer, Young T Hong, Guy Williams, Franklin Aigbirhio, James B Rowe, John T O'Brien

**Affiliations:** 1 Department of Psychiatry, University of Cambridge, Cambridge, UK; 2 Department of Clinical Neurosciences, University of Cambridge, Cambridge, UK; 3 Division of Anaesthesia, Department of Medicine, University of Cambridge, Cambridge, UK; 4 Department of Clinical Neurosciences, Wolfson Brain Imaging Centre, University of Cambridge, Cambridge, UK

**Keywords:** Tau, Neuroinflammation, [18F]AV-1451, [11C]PK11195, Positron emission tomography

## Abstract

**Introduction:**

Inflammation of the central nervous system is increasingly regarded as having a role in cognitive disorders such as dementia and depression, but it is not clear how such inflammation relates to other aspects of neuropathology, structural and functional changes in the brain and symptoms (as assessed via clinical and neuropsychological assessment and MRI). This study will explore these pathophysiological mechanisms using positron emission tomography (PET) which allows in vivo imaging of inflammation, amyloid and τ deposition, together with neuropsychological profiling, MRI and peripheral biomarker analysis.

**Methods and analysis:**

Using PET imaging of the ligand [^11^C]PK11195, we will test for increased neuroinflammation in vivo in patients with Alzheimer's disease, Lewy body dementia, frontotemporal dementia, progressive supranuclear palsy, late-onset depression and mild cognitive impairment, when compared to healthy controls. We will assess whether areas of inflammatory change are associated with amyloid and τ deposition (assessed using ^11^C-labelled Pittsburgh Compound B ([^11^C]PiB) and ^18^F-labelled AV-1451, respectively), as well as structural and connectivity markers found on MRI. Inflammatory biomarker analysis and immune-phenotyping of peripheral blood monocytes will determine the correlation between central inflammation and peripheral inflammation. Finally, we will examine whether central inflammatory markers seen on PET imaging are associated with global and domain specific cognitive impairments or predict cognitive decline over 12 months.

**Ethics and dissemination:**

The study protocol was approved by the local ethics committee, East of England—Cambridge Central Research Ethics Committee (reference: 13/EE/0104). The study is also Administration of Radioactive Substances Advisory Committee (ARSAC) approved as part of this process. Data will be disseminated by presentation at national and international conferences and by publication, predominantly in journals of clinical neuroscience, neurology and psychiatry.

**Strengths and limitations of this study**

- Multimodal deep phenotyping.
- Comparisons between diseases as well as with controls.
- Longitudinal neuropsychology data.
- Comparison of central and peripheral inflammation.
- Unable to assess causation.

## Introduction

Over 800 000 people in the UK have dementia^1^ for which there are many causes including Alzheimer's disease (AD), Parkinson's disease dementia (PDD), dementia with Lewy bodies (DLB), frontotemporal dementia (FTD) and progressive supranuclear palsy (PSP). There are also many more people with at risk states such as mild cognitive impairment (MCI) and late life depression (LLD). Since depression and dementia have many symptoms in common, including cognitive impairments and low mood, they may also share common neurobiological substrates.

The Neuroimaging of Inflammation in Memory and Related Other Disorders (NIMROD) project has been established to test the hypothesis that neuroinflammation plays a pivotal role in the pathogenesis of different forms of dementia and related disorders. Neuroinflammation describes the cellular and biochemical response to a variety of insults within the central nervous system (CNS) and includes the activation of microglia which are the resident macrophages of the CNS. Microglia provide the innate immune response to invading pathogens and also initiate the adaptive response through antigen presentation.^2^ [^11^C]PK11195, a positron emission tomography (PET) ligand that binds to the translocator protein (TSPO) which is upregulated on microglia in their activated state, allows the imaging of neuroinflammation in vivo. Other ligands that bind TSPO have been developed (these include [^11^C]PBR28, [^11^C]DPA713, [^11^C]DAC and [^18^F]DPA-714), however their use is complicated by intersubject variability in binding affinities to TSPO^3^ that does not affect [^11^C]PK11195, which remains the best characterised and most widely used ligand. Although affinity is lower than some second-generation ligands for TSPO, it has the advantage of a well-established robust protocol for synthesis and analysis and is less affected by genetic variations in affinity.

There is growing evidence for a link between inflammation and depression^4^ ^5^ and between inflammation and a broad range of neurodegenerative disorders. Such evidence of neuroinflammation is strongest in AD^6^ and includes neuropathological studies revealing evidence of brain inflammation and PET imaging displaying microglial activation in vivo^7–10^ which may well play a protective role in early disease.^11^ Animal studies in AD as well as old age have also found that drugs antagonising the Leukotriene pathway and thereby modulating inflammation have beneficial effects on cognition.^12^ ^13^ Similarly, PET imaging and pathology studies showing evidence of inflammation are emerging in DLB and PDD.^14–16^ In vivo inflammation has also been shown in MCI, however results in several studies were conflicting.^17^ In FTD^17^ ^18^ and PSP^19–21^ studies of inflammation are in their early stages but are suggestive of a possible increased rate of chronic neuroinflammation.

The deposition of insoluble, misfolded proteins, such as hyperphosphorylated τ or β amyloid, has long been recognised as a feature of neurodegenerative disease such as AD^22^ and frontotemporal lobar degeneration which includes FTD and PSP. More recently, analogous pathological accumulations of α-synuclein in PD,^23^ and TAR DNA binding protein 43 (TDP-43) in several forms of frontotemporal lobar degeneration and motor neuron disease,^24^ have been found. In some diseases, the degree of abnormal protein deposition correlates with clinical severity and progression, for example using Braak staging of pathology in AD.^25^

The ligands to image protein accumulations in vivo have developed greatly in the last decade. The first of these to make a major impact in research and clinical practice was the ^11^C-labelled Pittsburgh compound B ([^11^C]PiB) for β-amyloid, with ^18^F-labelled analogues (florbetaben, florbetapir and flutemetamol) being developed subsequently.^26^ A range of new compounds has recently been developed that bind to τ deposits. These include [^18^F]AV-1451,^27^ THK^28^ and PBB3 ligands.^29^ [^18^F]AV-1451 has been examined in postmortem studies of Alzheimer's pathology^30^ ^31^ with encouraging results supporting its use in vivo. Recently published work has correlated [^18^F]AV-1451 binding with Braak stage in typical AD^32^ and distribution of binding with focal AD syndromes.^33^ The extent and location of [^18^F]AV-1451 binding in vivo in MAPT mutation carriers correlated well with extent and distribution of pathology in the same subject at postmortem^34^ while statistically significant differences in amount and distribution of binding have been shown between a subject with a MAPT mutation and healthy older controls.^35^ There are concerns about ‘off target’ binding of [^18^F]AV-1451 to sites such as Neuromelanin^36^ and Choroid plexus,^37^ but these need verification. Much work still needs to be performed to determine specificity and sensitivity of the [^18^F]AV-1451 ligand, particularly in non-AD tauopathies such as FTD and PSP.

Here we use three PET ligands: [^11^C]PK11195, [^11^C]PiB and [^18^F]AV-1451 in patients with different forms of dementia, related disorders and controls. Using [11C]PK11195 PET, we will assess for microglial activation in vivo in all cohorts (ie, AD, MCI, FTD, LBD, PSP and LLD). Few studies have investigated microglial activation in FTD, LBD, PSP and LLD so this key aspect of our project is novel. We will also compare how the extent and pattern of microglial activation relates to clinical symptoms in each of these conditions, separately and across groups. For example, our study will assess whether increased microglial activation in the hippocampus is associated with increased memory symptoms or if visual hallucinations in DLB are associated with increased occipital lobe microglial activation.

In addition, all cohorts will be assessed for peripheral markers of inflammation and structural and functional abnormalities on MRI. This will include immune-phenotyping to analyse for changes within the adaptive immune system, as reflected by changes in T-cell subsets as well as serum inflammatory markers to identify changes in both arms of the immune system. Studies in AD have shown evidence of peripheral inflammatory changes,^38^ which may well be replicated in other cognitive disorders. Changes in CD4 cells have been recognised in PD, for example.^39^ ^40^ Structural, vascular and functional connectivity changes will be analysed using MRI. Both of these modalities will be compared to central microglial activation to determine if central inflammation is linked to either in the brain. The relevance of baseline biomarkers to clinical disease progression will also be tested through annual follow-up of participants.

Data obtained from [18F]AV-1451 and/or [11C]PiB binding (depending on the cohort) will be correlated with data from [11C]PK11195 PET imaging to look for areas of overlap between protein deposition and microglial activation. The relationship between microglial activation and symptomatology will be assessed in the context of any underlying increased protein deposition.

## Summary of research questions

1. Is there an increase in microglial activation in each of AD, DLB, PDD, PSP, FTD, LLD and MCI groups, compared to age-matched control participants?
2. Does the extent and pattern of any increased microglial activation in these disorders relate to:
  - key clinical symptoms, such as deficits in memory, attention, motor and visual symptoms and their relevant cortical areas in each cohort?
  - peripheral markers of neuroinflammation such as changes in serum cytokines and alterations in T-cell subsets when compared to participants in the control group?
  - structural changes on MRI, including atrophy, vascular changes and deficits in white matter tracts in relevant regions compared to control participants?
  - alterations in functional connectivity as measured using resting-state Blood Oxygenation Level Dependent (BOLD) fMRI compared to control participants?
  - the extent and pattern of increased brain amyloid deposition as assessed by [^11^C]PiB PET in DLB, PDD and MCI?
3. Is there increased τ deposition, as revealed by [^18^F]AV-1451 PET, in AD, PSP, FTD and MCI groups compared to age-matched control participants and does the extent and pattern of any increased τ deposition relate to the extent and pattern of microglial activation?
4. Does the distribution of binding differ between disease cohorts and from controls as would be predicted by known anatomical correlates of the clinical syndromes?
5. Does increased central microglial activation and/or changes in peripheral markers of inflammation predict subsequent clinical course, including future cognitive and functional decline?

## Methods and analysis

### Summary

**Figure 1:**
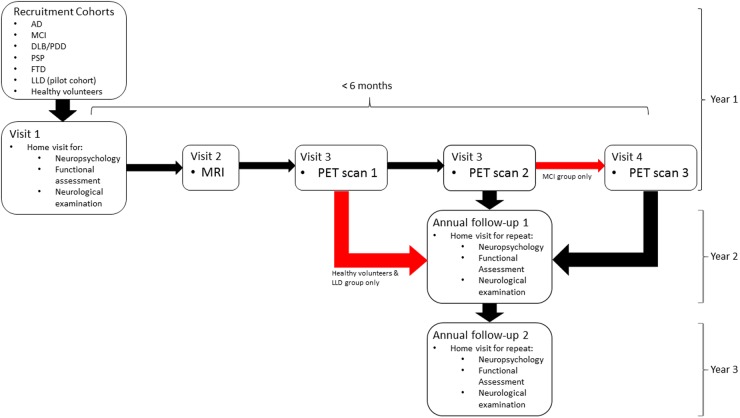
Flow chart illustrating participants' journey through the study. AD, Alzheimer's disease; DLB, dementia with Lewy bodies; FTD, frontotemporal dementia; LLD, late life depression; PDD, Parkinson's disease dementia; PSP, progressive supranuclear palsy.

The observational study protocol consists of neuropsychological assessment and neurological examination with annual reappraisal over 3 years (figure 1). These baseline assessments are followed by one visit for MRI and then one, two or three visits for PET depending on the cohort. A cerebrospinal fluid (CSF) substudy will be conducted in smaller numbers consented for this purpose.

### Participants, recruitment and selection

Patient participants are recruited from cognitive disorder clinics in neurology, old age psychiatry and related services at Cambridge University Hospital (CUH) and other trusts within the region including Cambridgeshire, Lincolnshire, Bedfordshire, Norfolk, Suffolk, Hertfordshire and Essex, where participants are willing to travel to Cambridge for imaging studies. Case registers held by the Dementias and Neurodegeneration specialty of the UK Clinical Research Network (DeNDRoN) and the Join Dementia Research (JDR) platform^41^ are other sources of participants. Control participants are recruited from regional healthy adults who have indicated a willingness to participate in dementia research via JDR or DeNDRoN. We also recruit interested, healthy friends and non-blood-related family members of patients.

Potential participants identified as above who show willingness to take part in the research are provided with information about the study in the form of a patient information sheet. Following a period of time to consider the information, a follow-up phone call is made to inquire as to their interest in participation and to ask for further information to ensure they are eligible to take part. An appointment is then made at the study premises or at their home to provide an opportunity to ask further questions and obtain formal written consent from the participant or, in cases where the participant does not have capacity, advice from an appropriate consultee in accordance with the Mental Capacity Act 2005 (England and Wales). Consent is for participation in the study and publication of findings.

### Eligibility criteria

Participants are to be included in the study if they are aged over 50 and have sufficient proficiency in English to allow standardised cognitive testing. All participants except controls must have a reliable informant who is able to complete questionnaires for informant-related scales and provide a background history in order to be included. Further specific inclusion criteria for each cohort are listed below under ‘Cohorts’. For the cohorts including participants with dementia (groups 2, 4 and 6 below), we have included only participants with mild-to-moderate dementia, as our experience is that severely impaired participants are highly unlikely to comply with the study protocol. Mild-to-moderate dementia are defined in this study as mini mental state examination (MMSE) >12, though those with language and/or semantic impairments (including the semantic variant of FTD), for which the MMSE is an unsuitable screening test, will be assessed using the Clinical Dementia Rating Scale,^42^ with a score of 2 or less indicating mild-to-moderate dementia.

### Exclusion criteria

Potential participants are excluded if they have a concurrent major psychiatric illness (except if this is depression in the LLD cohort) or if they have a contraindication to an MRI scan (such as a permanent pacemaker), are unable to tolerate an MRI (due to claustrophobia) or if they have a comorbidity that limits their ability to take part in the study. Participants with Parkinson's disease (except in the DLB and PDD cohorts) or previous head injury will also be excluded. Potential participants may also be excluded if they have atypical or focal parenchymal appearances on MRI which are not in keeping with their diagnosis. Systemic inflammatory disease is also an exclusion criterion, as are concurrent medications that may affect study assessments (eg, oral steroids).

### Cohorts

Participants are recruited into seven separate cohorts. Unless otherwise stated, our intention is to recruit a minimum of 16 participants (maximum 30) in each diagnostic group, so providing 80% power to detect an effect size of 1.0 between two groups. The cohorts will be:

1. Healthy control participants, defined as participants with MMSE scores >26 and with an absence of (i) regular memory symptoms, (ii) signs or symptoms suggestive of dementia or (iii) unstable or significant medical illnesses.
2. Participants with AD who meet the diagnostic criteria for probable AD as defined by National Institute on Aging-Alzheimer's Association workgroups on diagnostic guidelines for AD.^43^
3. Participants with MCI, defined as participants having an MMSE >24 but with memory impairment beyond that expected for age and education which does not meet criteria for probable AD and is not explained by another diagnosis.^44^
4. Participants with Lewy body dementia, meeting either the 2005 consensus criteria for probable DLB^45^ or the Movements Disorders Society clinical diagnostic criteria for PDD.^46^
5. Participants with progressive supranuclear palsy fulfilling the Litvan criteria, modified by a relaxation of the falls criterion to falls within 3 years, rather than 1 year, as suggested by the NNIPPS-PSP study group.^47^
6. FTD syndromes as defined clinically by Rascovsky *et al*^48^ and Gorno-Tempini *et al*.^49^
7. Five participants (to provide pilot data) with current or a recent diagnosis of LLD as determined by the DSM-IV criteria (American Psychiatric Association 2000).

### Overview of protocol

Once written consent has been provided, participants have a neuropsychological assessment using a test battery described in detail below. The battery is tailored to the cohort to which the participant belongs. All participants also undergo an initial clinical assessment, including the collection of clinical and demographic information (including medication, smoking, alcohol and education histories).

Participants then make between two and four visits for imaging depending on their cohort. All participants have an MRI scan. Healthy control participants will undergo one PET scan (either with [^11^C]PK11195 or [^18^F]AV-1451) as will participants in the LLD cohort ([^11^C]PK11195). MCI participants have three PET scans ([^11^C]PK11195, [^18^F]AV-1451 and [^11^C]PiB). Participants in all other cohorts will have two PET scans (for DLB cohort [^11^C]PK11195 and [^11^C]PiB, for AD, PSP and FTD cohorts [^11^C]PK11195 and [^18^F]AV-1451).

Venepuncture is carried out at the time of [^11^C]PK11195 imaging in all participants to measure peripheral inflammatory and other degenerative markers. Participants who provide additional consent will also have a lumbar puncture for analysis of CSF, measuring established and emerging candidate biomarkers of neurodegeneration.

Each participant undergoes repeat neuropsychological testing annually, for up to 3 years, to provide a longitudinal assessment of cognitive function. Further details of each of these stages are set out below.

### Initial clinical assessment, neuropsychological battery and informant questionnaires

At the initial visit, neuropsychological testing is undertaken using the battery given in table 1: *neuropsychological testing*. The neuropsychology battery differs slightly between cohorts because of the disease-specific domains we wish to examine. Clinical assessment is carried out either at the same visit or on a day of attendance for imaging. Neuropsychological follow-up using the same battery of tests is undertaken annually for up to 3 years from the date of initial assessment.

**Table 1:** Neuropsychological testing

Test name	Format	Purpose	Cohort
Clinical assessment			
UPDRS part III (motor subscale)^50^	Performed by study clinician	Measure of Parkinsonism (motor aspects)	All
Physical examination of eye movements	Part of physical examination performed by study clinician	Assessment of range and speed of eye movements	All
Frontal assessment battery^51^	Assessment tool completed by study clinician	Assessment of frontal lobe function	FTD, PSP
PSP Rating Scale^52^	Assessment tool completed by study clinician	Assessment of disease severity	PSP
Praxis battery	Part of physical examination performed by study clinician	Assessment of manual ideomotor and copying ability	All
Neuropsychological assessment			
Addenbrooke's cognitive examination—revised^53^	Researcher administered structured test	Multidomain cognitive screening tool	All
INECO frontal screening^54^	Researcher administered structured test	Assessment of frontal lobe function	All
Trails A and B	Researcher administered structured test	Assessment of executive function	All
Rey auditory verbal learning test	Researcher administered test of learning, recall and repetition of semantically unrelated words	Test of verbal episodic memory	All except PSP
Pyramids and palm trees	Researcher administered, two alternative, forced choice, picture based test	Assessment of semantic memory	All
Cambridge Neuropsychological Test Automated Battery (CANTAB)			
Simple reaction time^55^	Researcher administered computer task	Information processing speed	All
Paired associate learning^56^	Researcher administered computer task	Assessment of visual episodic memory and learning	All
Stockings of Cambridge^57^	Researcher administered, computer based spatial planning task	Test of frontal lobe function	All
Mental health questionnaires			
Hospital Anxiety and Depression Scale	A 14 item self-reported questionnaire	Assessment of symptoms of anxiety and depression	All
Geriatric Depression Scale	A 30 item self-reported questionnaire	Assessment of depressive symptoms	All
Montgomery-Asberg Depression Rating Scale^58^	A 10 item self-reported questionnaire	Assessment of severity of depressive symptoms	Controls, LLD
Informant questionnaires			
Cambridge Behavioural Inventory^59^	An 81 item carer-reported questionnaire	Assessment of several behavioural abnormalities in the everyday life including impulsivity and apathy	All except controls
Clinical Dementia Rating Scale^42^	A carer-reported numerical scale	Quantifying severity of dementia	All except controls
Bristol Activities of Daily Living Score	A 20 item carer-reported questionnaire	Measure of ability of person with dementia to carry out activities of daily living	All except controls
Neuropsychiatric inventory^60^	Researcher administered, carer-reported 13 item screening tool	Assessment of psychopathology in people with brain disorders	All except controls
Clinical Assessment of Fluctuating Confusion and Quality of Consciousness^61^	Researcher administered, carer-reported 9 item screening tool	Assessment of conscious level and degree of symptomatic arousal fluctuation	All except controls

FTD, frontotemporal dementia; INECO, Instituto de Neurologia Cognitiva; LLD, late life depression; PSP, progressive supranuclear palsy; UPDRS, Unified Parkinson's Disease Rating Scale.

### MRI imaging

MRI scanning will be carried out at the Wolfson Brain Imaging Centre (WBIC) using 3 T Siemens scanners. The following sequences are performed during the scanning protocol:

1. Three-dimensional structural high-resolution T1-weighted sequence examining for structural brain abnormalities (176 slices of 1.0 mm thickness, first echo time (TE)=2.98 ms, repetition time (TR)=2300 ms, flip angle=9°, acquisition matrix 256×240; voxel size=1×1×1 mm^3^).
2. Perfusion (pulsed arterial spin labelling) for blood flow (9 slices of 8.0 mm thickness, TE=13 ms, TR=2500 ms, acquisition matrix 64×64; voxel size=4×4×8 mm^3^, inversion time 1=700 ms, inversion time 2=1800 ms).
3. Diffusor tensor imaging (DTI) to obtain fractional anisotropy measures of white matter integrity and gross axonal organisation (63 slices of 2.0 mm thickness, 63 diffusion directions, TE=106 ms, TR=11 700 ms, b-value 1=0 s/mm^2^, b-value 2=1000 s/mm^2^, acquisition matrix 96×96; voxel size=2×2×2 mm^3^).
4. Susceptibility-weighted imaging (SWI) to identify microhaemorrhages, venous blood and iron deposition (40 slices of 2.0 mm thickness, TE=20 ms, TR=35 ms, flip angle=17°, acquisition matrix 256×240; voxel size=1×1×2 mm^3^).
5. High-resolution hippocampal subfield sequences carried out in coronal T2 for smaller structural changes in the hippocampus (20 slices of 2.0 mm thickness, TR=6420 ms, flip angle=160°, acquisition matrix 512×408; voxel size=0.4×0.4×2 mm^3^).
6. Resting state functional MRI with pulse and breathing monitored to examine ‘task-free’ functional brain connectivity (34 slices of 3.8 mm thickness, TE=13 ms, TR=2430 ms, flip angle=90°, acquisition matrix 64×64; voxel size=3.8×3.8×3.8 mm^3^, duration 11 min and 5 s).
7. T2 Fluid Attenuated Inversion Recovery (FLAIR) for characterising periventricular lesions adjacent to the sulci and white matter lesions and hyperintensities (75 slices of 2 mm thickness, TE=132 ms, TR=12 540 ms, flip angle=120°, acquisition matrix 256×256; voxel size=0.9×0.9×2 mm^3^).

All images will be examined by a Consultant Radiologist at CUH to exclude unexpected brain abnormalities in recruits. Participants with significant abnormalities will be excluded from the study.

### PET imaging

The radiotracers are produced at the WBIC Radiopharmaceutical Chemistry laboratories. Both of the ^11^C-labelled compounds as well as [^18^F]AV-1451 will be produced using the GE PETtrace cyclotron, a 16 MeV proton and 8 MeV deuteron accelerator. [^11^C]PK11195 will be prepared using the ‘Disposable’ synthesis system or GE TRACER laboratory FX-C module, whereas [^11^C]PiB will be prepared using the GE TRACER laboratory FX-C module. The production of [^18^F]AV-1451 is based on the synthetic methods developed by Avid Radiopharmaceuticals and modified to use the GE TracerLab FX-FN synthesiser at WBIC.

#### [^11^C]PK11195 PET

500 MBq [^11^C]PK11195 is injected intravenously over 30 s at the onset of a 75 min scan, with emission data subsequently reconstructed into 55 contiguous time frame images for kinetic analysis with the simplified reference tissue model.^62^

#### [^11^C]PiB PET

550 MBq of [^11^C]PiB is injected as a bolus and a PET scan is performed 40–70 min postinjection, providing image data suitable for subsequent standardised uptake value ratio (SUVR) analysis.

#### [^18^F]AV-1451 PET

370 MBq of [^18^F]AV-1451 is injected intravenously over 30 s at the onset of a 90 min scan, with emission data subsequently reconstructed into 58 contiguous time frame images for kinetic analysis with the simplified reference tissue model.

### Blood samples

Eighty millilitres of blood is drawn at the time of [^11^C]PK11195 PET scan. Basic inflammatory markers are tested at the time of the sample with the remaining blood being centrifuged to produce serum and plasma samples to be stored at −80°C for future testing of inflammatory and neurodegenerative markers. A subgroup of participants also have blood immunophenotyping based on the availability of the technique to the study. These participants will have blood samples analysed on the same day using flow cytometry for immune-phenotyping.

### CSF samples

For those who consent to the additional CSF substudy, CSF samples are obtained on a separate study visit. The samples are obtained by a lumbar puncture and stored at −80°C for future testing of inflammatory and neurodegenerative markers.

### Analysis

Primary analysis of the PET data will compare each subject group to controls based on [^11^C]PK11195 non-displaceable binding potential (BP_ND_) and [^11^C]PIB SUVR and/or [^18^F]AV-1451 BP_ND_; BP_ND_ and SUVR provide estimates of the density of ligand binding sites, with BP_ND_ being a more specific measure. Likewise, MRI data from patient groups will be compared to that of controls and across groups. Analysis of PET and MRI data use voxel-based and region of interest approaches. We will also explore surface-based PET approaches in order to improve signal-to-noise ratio in [^11^C]PK11195 analysis.^63^ Blood inflammatory markers and CSF measures of inflammation and τ, phosphor-τ, Aß and α-synuclein will be compared using standard parametric methods. Depending on initial analysis, between group comparisons of imaging, cognitive and biomarker data will be undertaken.

Neuropsychological test scores will be converted to domain z scores for correlational analysis with imaging data. Repeat measures will be normalised to control scores at the relevant time point, and annualised change scores correlated with baseline imaging changes to determine predictors of decline, using univariate and multivariate approaches as appropriate to determine key predictors. Genetic analysis will be used to identify known alleles associated with disease and for inflammation and exploratory analyses.

## Current status and dissemination

The study is Administration of Radioactive Substances Advisory Committee (ARSAC) approved. The study is already underway with active recruitment and data collection. This will continue until Spring 2017. Early analyses are on-going and have been presented at recent national and international conferences. Data will continue to be disseminated by presentation at national and international conferences and by publication, predominantly in journals of clinical neuroscience, neurology and psychiatry. The cohort is part of the MRC funded Dementias Platform UK collaboration.
